# A Single-Team Case Study of Corrective Exercises for Upper-Extremity Injuries and Movement Dysfunction in Collegiate Swimmers

**DOI:** 10.3390/sports13100349

**Published:** 2025-10-03

**Authors:** Kristen G. Quigley, Madison Fenner, Philip Pavilionis, Nicholas G. Murray

**Affiliations:** Department of Kinesiology, School of Public Health, University of Nevada, Reno, NV 89557, USA; kristenquigley@unr.edu (K.G.Q.); madisontaylor@unr.edu (M.F.); ppavilionis@unr.edu (P.P.)

**Keywords:** swimming, prevention, exercise, student-athletes

## Abstract

Swimming research has determined that rounded shoulders, forward head, and scapular dyskinesis are common imbalances that may lead to injury without correction. This case study aimed to evaluate a preventative exercise program designed to reduce injuries, correct postural deviations, and improve shoulder function over one collegiate swimming season. Twenty female NCAA Division I swimmers (average age = 21.6 ± 1.3 years) participated over 25 weeks, completing pre-, mid-, and post-season assessments of injury rates, shoulder range of motion, and stability using standardized tests. Injuries were included as diagnosed and reported by an athletic trainer. Testing included internal rotation, external rotation, the Hawkins-Kennedy test, Neer’s sign, Sulcus sign, and the Closed Kinetic Chain Upper-Extremity Stability Test (CKCUEST). Compared to the season prior with no intervention, swimmers who completed the program were 44% less likely to sustain an upper-extremity injury, as assessed from the CKCUEST scores (*p* < 0.01 for all metrics), shoulder internal rotation (*p* < 0.01 for both shoulders), and total range of motion (*p* < 0.01 for both shoulders). These findings suggest that a targeted corrective exercise program can effectively reduce injury rates and improve shoulder mobility and function in collegiate athletes. The interpretation of these results is limited by the study’s non-randomized design and absence of a control group.

## 1. Introduction

Shoulder injuries account for 40–91% of all swimming related injuries [1,2,3] and affect 66% of all Olympic swimmers [4]. High training volume and repetitive overhead motions contribute to fatigue and overuse, with 21% of swimmers at the 2015 World Championships reporting regular analgesic use, and 38% modifying training due to pain [5].

Shoulder injuries in swimmers are linked to factors such as pectoralis minor tightness, decreased core endurance [6], poor stroke mechanics, increased glenohumeral laxity, and cumulative training exposure [7]. Long-term swimming may lead to reduced mechanical properties in the supraspinatus [8] and biceps tendon, increasing the risk of impingement syndromes [4]. These risk factors often result in postural malalignments, muscular imbalances, and altered scapular kinematics [9,10], collectively termed “swimmer’s shoulder”, which encompasses rotator cuff tendinitis, shoulder instability, and impingement [9,10]. Specifically, swimmer’s shoulder describes the adaptative changes in the rotator cuff, bursa, labrum, and capsule to protect the shoulder from overuse injuries [4].

Swimming-related epidemiology is at the forefront of research efforts, with these reaching a consensus that rounded shoulder posture, forward head posture (FHP), and scapular dyskinesis are common postural imbalances among swimmers that may lead to injury [7,9,10,11,12]. Rounded shoulder posture is often caused by the protracted position of the scapula, caused by the imbalance of shortened pectoralis muscles and a lengthened middle trapezius [7,10]. Research has also pointed to a need for strengthening the serratus anterior, lower trapezius, and rhomboids, which are associated with scapular dyskinesis [7,11,12]. With scapular dyskinesis in particular, neuromuscular activation patterns differ in swimmers with shoulder pain, causing greater activation of the upper trapezius, serratus anterior, and latissimus dorsi [13]. Glenohumeral Internal Rotation Deficit (GIRD), present in 18% swimmers [14], arises from a tight posterior capsule [15] and is defined as an internal rotation (IR) discrepancy of 20° or more between shoulders. IR loss is often accompanied by gains in external rotation (ER), compensating for the anterior shift in the humeral head [15]. Finally, FHP is usually accompanied by the shortening of the upper trapezius, levator scapulae, and various spinal stabilizers [10]. Research has associated FHP with shoulder pain due to the decrease in the available upward rotational range of motion of the scapula, which is a known cause of shoulder impingement [10,16]. While epidemiology data is informative, it does little to provide coaches with methods to counteract the effects of high-volume swimming training. For this reason, this case study focuses on the implementation and effect of a corrective exercise program within a single National Collegiate Athletic Association (NCAA) Division I women’s swim team across one season.

Studies have applied epidemiology data to create rehabilitation programs for swimmers that use a multifaceted approach of strengthening and range-of-motion (ROM) exercises [7,9,10,11]. Recommended exercises varied; however, the programs mainly utilized scapular stabilization and shoulder ER exercises [7,9,10,11]. Chin tucks, the push-up plus, external rotation, and row variations were among the most commonly recommended exercises [7,9,10,11]. Some studies described exercises for known dysfunctions without assessing efficacy [7,9]; however, those that implemented corrective programming report significant improvements in posture [10] and a decrease in excessive glenohumeral IR after 6–8 weeks [11]. These results were determined by reductions in perceived pain and/or improvements on static postural and movement screenings [10,11]. Both studies used relatively short program lengths, such as 6 [11] or 8 [10] weeks. The conference collegiate swimming season is about 26 weeks; therefore, neither study encompassed the length of an entire season. A short program repeated continuously without much exercise progression may lead to reduced benefits or a lack of interest from the athletes. It is also likely that the repetitive nature of swimming over a season requires a more consistent exercise program, not a short intervention. While not always mentioned in similar studies [7,9,10,11], it is also important to consider retraining altered neuromuscular firing patterns through corrective exercises, particularly with the serratus anterior, latissimus dorsi, and upper trapezius [13].

Although much research investigates swimming-related injuries on a population level, there is limited evidence drawn from detailed, real-world intervention within a single-team context. This manuscript presents a case study examining the implementation and outcomes of a corrective exercise intervention conducted exclusively within one NCAA Division I women’s swim team. For these reasons, this case study aimed to evaluate the efficacy of a season-long, novel, preventative, upper-extremity exercise program in reducing injuries, correcting postural deviations, and addressing range-of-motion deficits among collegiate swimmers over a single NCAA season using injury reports, movement screens, and clinical orthopedic testing. This program aims to be accessible in all levels of participation and is practical for programs with restricted resources. Based on prior research [14,15], it is hypothesized that most of the swimmers in this study will exhibit positive indicators of GIRD, as exhibited by shoulder range-of-motion (ROM) assessments. It is additionally hypothesized that the exercise program will reduce injuries by improving shoulder range of motion and symptoms of impingement. This case study design was chosen to generate in-depth insights that can be translated directly into practice for similar NCAA programs, although the findings are not intended for broad generalization.

## 2. Materials and Methods

### 2.1. Participants

Twenty female NCAA Division I swimmers (average age = 21.6 ± 1.3 years) participated in this case study during the 2024–2025 conference swimming season. The number of participants was obtained from a convenience sample based on the number of athletes on the respective university’s swim team. The frequency of upper-extremity injuries during the season prior (2023–2024) was collected at the same NCAA institution and included data from 20 Division I female swimmers (average age = 20.0 ± 1.3 years). The 2023–2024 cohort was only used as a reference for injury rates at the respective institution before the intervention of the corrective exercise program. Of the 20 athletes in the 2024–2025 cohort, 15 were included in the 2023–2024 injury dataset.

Participant age, height, and weight were recorded prior to the start of the season. Participants were included in this study if they were an eligible member of the respective swimming program. Participants were excluded due to any illness or injury, such as a concussion, that prevented them from completing either the laboratory assessments or the preventative exercise program in its entirety. All participants agreed to the study verbally and through a written consent form. This study was approved by the Institutional Review Board of the respective university and conducted in accordance with the Declaration of Helsinki.

From the previously mentioned criteria, 2 participants (10% of the recruited sample) were excluded for not completing all the exercise sessions due to an unrelated illness. An additional 2 participants refused to complete the Closed Kinetic Chain Upper-Extremity Stability Test (CKCUEST) due to pain during the first trial from pre-existing wrist injuries; however, they were only excluded from the CKCUEST analysis. After exclusionary criteria was applied, 16 participants completed the CKCUEST, and 18 participants completed every component except for the CKCUEST.

### 2.2. Study Variables

This study included several variables of shoulder stability, function, and pathology. First, this study conducted injury tracking of all upper-extremity injuries. This study also included ROM measurements for both the left and right shoulder that produced the following variables: IR, ER, and TRM. The CKCUEST was performed, resulting in a tap count score, normalized performance score, and normalized power score. Finally, this study recorded the incidence of positive indicators of impingement using the Hawkins-Kennedy test, Neer’s sign, and Sulcus sign for both shoulders. All measurements were conducted at three timepoints: PRE (prior to the start of the season, or week 1), MID (after the midseason competition, or week 13), and POST (after the conference championships, or week 26).

### 2.3. Procedures: Injury History and Tracking

Unique upper-extremity injuries from the championship season prior (2023–2024) were collected using electronic medical records (Table 1), including only those diagnosed by the certified athletic trainer (ATC) assigned to the respective team. The ATC assigned to the team had over 30 years of experience in the field, working only with overhead athletes specifically. Injuries were included in the medical records only if they led to the athlete seeking treatment from the ATC and/or requiring modifications during training. All injuries from the 2023–2024 season were cleared by the ATC prior to the start of the 2024–2025 season. During the 2024–2025 season, all uniquely diagnosed upper-extremity injuries were similarly recorded. While many kinds of injuries were included in the reports, this study only analyzed upper-extremity injuries (only shoulder and elbow; no wrist injuries reported) to assess the impact of the exercise program. Any injury involving the upper extremity was included; therefore, the included forms of injury were biceps tendinitis, subacromial bursitis, shoulder inflammation, rotator cuff strains, and trapezius strains. All injuries in this study were unilateral and deemed a swimming-related injury by the team’s ATC.

### 2.4. Procedures: Dynamic Assessments

The CKCUEST was used to assess shoulder functional performance, as it is a validated, low-cost test with intraclass correlation coefficients (ICCs) ranging from 0.77 to 0.92 for the mean number of touches and 0.80 to 0.94 for the normalized score in healthy young adults [15]. The CKCUEST has been used to evaluate shoulder dysfunction in previous studies [15,16,17,18,19], and this study evaluated changes in CKCUEST scores throughout the swimming season. Following prior research that used the CKCUEST in females, two pieces of tape were applied to the ground at a distance of 36 inches, or 91.44 cm [19]. The athlete was in a push-up position, with their hands on top of the tape. The athlete was instructed to remain in the push-up position while they alternatively tapped the top of the opposite hand for 15 s [19]. Following prior research that performed the CKCUEST in swimmers, the test was performed repeated four times in total, with a 45 s rest in between repetitions [19]. The first repetition served solely as an acclimation trial, and the average touch count from the other three trials was inputted into Equations (1) and (2) to measure performance and power [19]. CKCUEST power and normalized performance scores from the PRE, MID, and POST timepoints were compared.

Performance = (average touch count)/height(1)

Note: Height will be measured in inches.

Power = (((weight) × 0.68) × average touch count)/15(2)

Note: Weight will be measured in kilograms.

### 2.5. Procedures: Range-of-Motion (ROM) Testing

A universal goniometer was used to quantify each athlete’s passive internal and external range of motion at the shoulder. The goniometric measurements were collected at PRE, MID, and POST by the team’s ATC. A goniometer can provide joint measurements to the nearest degree and has established good reliability across a wide array of studies; however, its reliability has never been established for swimmers specifically [19,20,21]. The swimmers were in a supine position on a training table, with shoulder abduction at 90 degrees and elbow flexion at 90 degrees to stabilize the scapula. The goniometer was placed perpendicular to the floor, aligned with the axis of the olecranon, and the movable arm was aligned with the styloid process of the ulna to follow rotational movement [19]. GIRD testing is typically performed in overhead athletes who perform unilateral movements; however, as swimming is a bilateral sport, the results of the ROM tests were used to evaluate the swimmers for total rotational shoulder motion (TRM). TRM states the sum of IR and ER should not exceed 187 degrees [20]. The absolute value of difference in IR and ER between shoulders was also calculated (Equation (3)), along with the difference in TRM between the shoulders (Equation (4)). No form of inter-rater or intra-rater reliability was calculated.

Difference in IR: IR difference = |Left Internal-Right Internal|(3)

Note: The same equation is applied to external rotation (ER).

Difference in TRM: TRM difference = |Right TRM-Left TRM|(4)

### 2.6. Procedures: Clinical Testing

Clinical testing of the shoulder was performed to identify if any swimmers were suffering from shoulder impingement syndrome. The Hawkins-Kennedy test, Neer’s sign, and sulcus sign were all performed by the team’s ATC. Neer’s test required the clinician to stabilize the swimmer’s scapula while the other hand raised the swimmer’s arm into full flexion [20,22]. A positive Neer’s test means the patient experienced pain with the movement, as greater tuberosity is impinged against the anterior acromion [20,22]. The Hawkins-Kennedy test required 90 degrees of shoulder flexion, followed by forcible IR of the shoulder. A positive Hawkins–Kennedy test was determined by pain, caused by driving the greater tuberosity under the coracoacromial ligament [20,22]. Both Neer’s and Hawkins–Kennedy tests mimic motions of the freestyle pull; therefore, positive test results on these two tests likely indicates pain while swimming [23]. In addition, a sulcus sign test was performed [4,12]. A sulcus test demonstrates inferior ligamentous laxity of the shoulder [4]. Prior research suggests swimmers often have a positive sulcus test [12] and that a positive sulcus test can demonstrate an increased risk for shoulder impingement and rotator cuff tendinosis [4].

### 2.7. Procedures: Corrective Exercise Program

The corrective exercise program aimed to address injury rates and movement dysfunctions seen in swimmers [10,11,24,25,26,27]. The 15 min long exercise program created in this study was performed twice a week at the start of an aquatic training session throughout the entirety of the 25-week season. The progression of exercises was broken into phases that matched the flow of the respective team’s season, which is a common schedule across Division I swimming (Table 2). The duration of the program was 26 weeks, as the pre-season testing was completed during the first week of training (week 1) and the post-season testing was completed the week after the conference championships (week 26) (Table 2). The NCAA swimming season is typically viewed as two halves; therefore, this study allotted each half a unique set of three phases of corrective exercises. As the intensity of training increases during the second half of the season, an emphasis on stretching and myofascial release techniques was included in the second half. The corrective exercise program is available in Table 3.

Equipment was limited to light dumbbells and resistance bands to increase accessibility, as both items are low cost and are easy to store. The athletes in this study completed an additional 3 strength and conditioning sessions on land a week, which were not included in this study. The corrective exercise program, as well as the three testing timepoints, took place during the team’s training hours; meaning session attendance was not an issue. One member of the research team was also able to supervise all sessions and provide feedback when the athletes completed the exercises incorrectly.

### 2.8. Statistical Analysis

All statistical analyses were conducted using SPSS (IBM SPSS Statistics for Windows; Released 2022; Version 29.0; IBM Corp., Armonk, NY, USA). All data were evaluated for skewness and kurtosis to determine which statistical analyses best suited the data. Due to the small sample size of this study, effect sizes in the form of a Cohen’s *d* were calculated when possible. Per research from Fritz et al. (2024), the levels of effect size considered in this study were small (*d* < 0.20), medium (*d* = 0.50), and large (*d* > 0.80) [28].

Of all collected data, only the incidence of GIRD, along with the Hawkins–Kennedy test, Sulcus sign, and Neer’s sign pain responses, was non-parametric. As the data were binary and collected three times, Cochran’s Q test was selected to determine if the proportion of positive indicators of each clinical test improved.

All goniometric measurements (IR, ER, and TRM) and CKCUEST outcomes (tap count, power and performance) were parametric and analyzed using separate repeated measures analysis of variances (ANOVAs). To analyze significant time effects, post hoc testing in the form of Bonferroni corrections were performed to evaluate significance among pairwise timepoint comparisons.

The total number of upper-extremity injuries (i.e., sum of elbow and shoulder) were evaluated using a relative risk analysis. This method was chosen as it calculates the probability of an injury occurring in the untreated group (2023–2024) versus the intervention group (2024–2025) (Equation (5)). The probability of injury during each season was calculated by dividing the total number of upper-extremity injuries by the total number of athletes on the team.

Relative risk (RR): RR = ((Probability of injury during 2023–2024 season))/((Probability of injury during the 2024–2025 season))(5)

## 3. Results

### 3.1. Injury History and Tracking

Of the 20 swimmers included in the 2023–2024 injury data, 8 (40%) experienced a shoulder injury, and 0 experienced an elbow injury, resulting in 8 total upper-extremity injuries. During the 2024–2025 season, which was the timeframe of the preventative exercise program, 3 of the 18 swimmers (16.67%) incurred shoulder injuries, and 1 (5.56%) incurred an elbow injury, resulting in 4 total (22.22%) injuries. The relative risk was reduced by 44%, meaning swimmers who participated in the preventative exercise program were 44% less likely to experience a UE injury (Table 1).

### 3.2. Dynamic Assessments

A repeated measures ANOVA revealed significant improvements in the mean normalized CKCUEST performance scores (F(2,14) = 14.12, *p* < 0.001), and Bonferroni corrections revealed significant differences between PRE and MID (*p* < 0.001), MID and POST (*p* < 0.001), and PRE and POST (*p* < 0.001). Mean CKCUEST power scores (F(2,14) = 14.86, *p* < 0.001) also significantly differed, with Bonferroni corrections revealing significant differences between PRE and MID (*p* < 0.001), MID and POST (*p* < 0.001), and PRE and POST (*p* < 0.001). An additional repeated measures ANOVA also found significant improvements throughout the study on the mean number of touches of the CKCUEST (F(2,14) = 14.28, *p* < 0.001). Bonferroni corrections for the number of touches showed significant differences between PRE and MID (*p* < 0.001), MID and POST (*p* < 0.001), and PRE and POST (*p* < 0.001). The means, standard deviations, and effect sizes from each CKCUEST measure are in Table 4.

### 3.3. Range-of-Motion (ROM) Testing

Of the nine individual repeated measures ANOVAs run on the goniometric measurements, only four produced significant results. The IR of both the right shoulder (F(2,16) = 34.11, *p* < 0.001) and left shoulder (F(2,16) = 61.36, *p* < 0.001) improved significantly across the three timepoints. Further probing through Bonferroni corrections for the right shoulder showed significant improvements from PRE to MID (*p* = 0.003), PRE to POST (*p* < 0.001), and MID to POST (*p* < 0.001). On the left shoulder, Bonferroni corrections revealed significant increases from PRE to POST (*p* < 0.001) and MID to POST (*p* < 0.001), while near significance was found from PRE to MID (*p* = 0.05). The TRM improved significantly for both the right shoulder (F(2,16) = 40.50, *p* < 0.001) and left shoulder (F(2,16) = 22.97, *p* < 0.001). For the right shoulder, Bonferroni corrections suggested improvements were seen from PRE to POST (*p* < 0.001) and MID to POST (*p* < 0.001), and no improvements were seen from PRE to MID (*p* = 0.35). Cochran’s Q analysis revealed no significant differences (*p* = 0.45) in the incidence of GIRD across PRE, MID, and POST. All scores and results can be found in Table 5.

### 3.4. Clinical Testing

A Cochran’s Q analysis found no significant improvements in the number of swimmers reporting pain with the Hawkins-Kennedy (left: *p* = 0.51, right: *p* = 0.61) and Neer’s (left: *p* = 0.61, right: *p* = 0.37) sign test in both shoulders. As no swimmers had a positive Sulcus test in either shoulder at any timepoint, the data was not analyzed further. All results can be found in Table 6.

## 4. Discussion

This case study examined the efficacy of a preventative exercise program at reducing injuries, correcting postural deviations, and addressing ROM deficits in female collegiate swimmers over a single season. This case study of a single NCAA Division I swim team demonstrates the feasibility and potential benefits of sustained corrective exercise programming in reducing upper-extremity injuries. The case study format allows for nuanced analysis of team dynamics and intervention implementation not captured in multi-site or survey research. A major finding was that the swimmers who participated in the corrective exercise program were 44% less likely to experience an upper-extremity injury compared to swimmers from the season prior. Contrary to the first hypothesis, only 16.7% of athletes showed signs of GIRD at any one timepoint (post-season). The program partially supported the second hypothesis, improving IR, TRM, and CKCUEST scores but not ER, ROM discrepancies, or shoulder impingement rates.

An impactful finding of this team-based case study was that the respective swimming team had a 40% risk probability of incurring a shoulder injury during the 2023–2024 season, which reduced to 22% during the 2024–2025 season. The risk of upper-extremity injury was reduced by 44% between seasons, suggesting the corrective exercise program was effective at reducing injuries. During both seasons, the swimmers completed an identical schedule of 10.5 h of aquatic exercise a week, coupled with 3 h of weight training. The volume of yardage swum or weights lifted was not recorded, which is an inherent limitation; however, it can be assumed that the workload between the seasons was similar, as the team goals had not changed. Additionally, the coaching staff and support staff (strength coach, ATC, etc.) were identical for both seasons, reducing the likelihood of significant differences between the seasons. The 44% risk reduction seen between seasons is a considerable difference that may be due to the exercise program, but no conclusions can be concretely drawn until future research replicates the program used in this study.

While the injury risk decreased, the rate of positive impingement results did not significantly change (Table 6). The number of swimmers with a positive Hawkins-Kennedy test was bilaterally highest mid-season (*n* = 4) and lowest post-season (*n* = 2); however, this result was not significant, perhaps due to the small sample size. A study by Fritz et al. (2012) found that 23.9% of masters swimmers experienced pain with the Hawkins-Kennedy test [28]. Despite collegiate swimmers likely having a higher training volume, the highest incidence of a positive Hawkins-Kennedy result was a bilateral rate of 22.2% (mid-season). The higher rate of impingement of masters athletes may be due to age-related factors and not swimming [29]. The lack of significant improvement in the rate of positive Hawkins-Kennedy results suggests that the exercise program was not able to correct the underlying pathology that causes pain.

This Division I women’s case study had 0 collegiate swimmers present with a positive sulcus sign in either shoulder at all three timepoints, which is significantly different than in prior studies. A study by Rodeo et al. (2016), using the sulcus sign to evaluate the relationship between shoulder laxity and tendinitis of the biceps, supraspinatus, and rotator cuff, in Olympic swimmers found that 98% of the swimmers studied had a positive sulcus sign [4]. Generally, Olympians engage in more rigorous training than the NCAA athletes in this study; however, future research should reexamine the incidence of a positive sulcus sign collegiate in swimmers.

Neer’s test was positive for 1 of the 18 athletes (5.56%) at some of the timepoints (Table 4). A 2018 study found that 37.3% Egyptian swimmers aged 12–25 years had a positive Neer’s sign [30], but as the athletes in this study were significantly older and were training at a more elite level, a higher rate of positive Neer’s results was expected. Considering the clinical tests results together, it seems positive that the swimmers in this study have lower impingement rates than swimmers in other studies. No study used similar populations, which may be what led to this discrepancy. As it relates to the exercise program, these results do not support the notion that the program reduced the prevalence of shoulder impingements. That said, as training became more intense throughout the season, it could be considered a positive result that the rate of positive tests did not increase as the training load increased. Considering the fact that injury rates decreased, perhaps the exercise program mitigated the escalation of impingements to injury but was not effective enough to alter the underlying mechanism of impingement.

Prior research found shoulder IR for collegiate swimmers to be 50–65° [21,31], which was not supported by this study. The lowest mean value of IR in this case study was 67.33 ± 10.69° on the right side at the pre-season timepoint, which is larger than in prior studies. It is important to note that one comparable study measured active ROM [31], and one measured passive ROM [21], the latter of which was used in this study. For both the right and left shoulders, IR improved significantly across the three timepoints (Table 5). Further probing of the significant results revealed that the improvements were seen from PRE to POST and MID to POST, while no significant changes occurred between PRE and MID. This may be due to the implementation of additional stretching exercises from MID to POST, whereas the corrective exercise program from PRE to MID mainly focused on strengthening (Table 3). The stretching exercises focused on the pectoralis muscle, which is a primary muscle in IR and is known to be tight in swimmers [6]. Another significant finding was the improvement in right and left TRM across the three testing timepoints (Table 5). Through Bonferroni corrections, it was revealed that the significant changes occurred from MID to POST and PRE to POST, with no significant improvement from PRE to MID. Contrarily, despite the numerous ER exercises included in the program, ER did not improve throughout this study. The lack of ER improvement suggests that the differences in TRM were solely due to improvements in IR (Table 5). While the swimmers in this study (minimum of 67°) started with a greater IR than in previous studies (30–40°) [15,21,31], the improvement across both shoulders suggests that the exercise program was successful at improving IR at the shoulder. The increased IR and TRM experienced by the swimmers may have contributed to the reduced injury rates seen in this study; however, future work should increase both IR and ER evenly to determine if that has a greater effect on injury rates.

It has been suggested that 18% of elite adolescent swimmers are at risk of developing GIRD [14]. In this team-based case study, only one (5.56%) swimmer had GIRD at PRE and MID, while three (16.67%) displayed signs of GIRD at POST, which was an insignificant increase (Table 5). These values are lower than the 2024 study [14], which may be explained by assuming that the adolescent athletes in the prior study were training at a lower volume. Another study of adolescent swimmers in 2009 found an IR discrepancy of 12 ± 6.8° between limbs, which is not classified as GIRD [31]. That study hypothesized that the loss of IR was due to an anterior shift in the humeral head and impingement of the shoulder [31]. The present study found mean IR discrepancies of 10.00 ± 5.62° at PRE, 6.50 ± 7.21° at MID, and 12.56 ± 8.86° at POST, similar to the 2009 study. Overall, the IR discrepancies and incidence of GIRD in this study are similar to prior research, despite the implementation of a novel preventative exercise program. As the exercise program did not significantly combat these imbalances, yet still reduced injuries, our findings suggest that injury reduction in this context may not be solely dependent on IR asymmetry or GIRD. This finding challenges prior assumptions that GIRD and IR asymmetry leads to injuries in swimmers, highlighting the need for a greater understanding of the mechanisms underlying injury prevention, as other factors may also be playing a critical role.

Among the final metrics calculated in this case study, this study observed significant improvements in all metrics of the CKCUEST throughout the season (Table 6). As the CKCUEST serves as a measure of shoulder dysfunction and strength, the improvements in this study suggest that the exercise program mitigated shoulder dysfunction and increased shoulder strength. Relating this to the reduced injury risk, the increased shoulder strength the swimmers experienced throughout the season may have aided in mitigating shoulder injuries. A study by Soares et al. (2023) compared CKCUEST scores from injured and non-injured swimmers (average age = 20 ± 7.9 years), training about 8–10 h a week [19]. The study found that the non-injured swimmers had an average touch count of 30.75 ± 3.8, average normalized performance score of 0.44 ± 0.05, and average power score of 222.4 ± 47.7 [19]. The comparable data from this study ranged from 24.10 to 28.96 for mean touch count, 0.35 to 0.43 for mean normalized performance, and 76.00 to 91.14 for mean power. While this study featured NCAA swimmers training twice the amount of those in the 2023 study, it is unclear why the power values have a large discrepancy. Both studies used same method of calculating power values, making it unclear why the values are wildly different. The present study did have moderate effect sizes for normalized performance (*d* = 0.67), power (*d* = 0.68), and touch count (*d* = 0.67), meaning this data may have practical significance. The prior study did not report effect sizes of any kind for comparison [19]. The improvement seen in the present study are promising; however, the discrepancies with the 2023 study suggests that more research on CKCUEST scores in swimmers is needed. As a single-team case study, the results are highly relevant to coaches and trainers working in similar settings, though external validity is limited. The findings serve as a foundation for future controlled or multi-site studies.

### Limitations

This study was limited by several factors. First, this study did not account for training volume or the style of training completed by the athletes. While NCAA athletes are subject to a controlled maximum number of training hours, it would have been beneficial to track the training hours completed by the athletes. Additionally, this study would have benefited from examining the strength program completed by the swimmers, as that program may have overlapped with the corrective exercise program. The strength program may also have contributed to the improvements seen in ROM or the CKCUEST scores, which is a limitation to the exercise program presented in this study. Further, while the sessions were supervised by a research team member who intervened with corrections as necessary, it is possible that the athletes still missed repetitions or poorly performed certain exercises, making exercise execution a possible limitation of this study. Third, the population of this study was limited to a small (*n* = 18) group of female swimmers. Some research used female-only cohorts [11], and there are more female Division I swimming programs; however, an ideal study would have used a larger, co-ed cohort. This study would have benefited from including a randomized control group for comparisons. While most single gendered swimming programs are not large enough to have a robust control and experimental group, this should be considered in future work. In line with needing a larger study population, this study was subject to reduced statistical power on the Cochran’s Q analyses, and thus an increased risk of Type II error. Finally, there may also have been psychological or motivational factors that influenced the injury rates or effort on the corrective exercises.

## 5. Conclusions

In summary, this case study demonstrates that a season-long corrective exercise program may reduce upper-extremity injury risk by 44% and improve shoulder mobility in collegiate swimmers, although larger, controlled studies are needed to confirm these findings. Additionally, the program was effective at increasing IR of the shoulder, increasing TRM of the shoulders, and improving CKCUEST scores with this team of female collegiate swimmers. As a case study, these results are specific to one collegiate swim team and should be interpreted in that context. The findings provide valuable insight into the potential benefits of targeted corrective exercises, but generalizability is limited.

## Figures and Tables

**Table 1:** Frequency (*n*) of reported athletic injuries from the 2023–2024 and 2024–2025 athletic seasons as diagnosed by a certified athletic trainer (ATC).

Season	Shoulder	Elbow	Risk Probability
2023–2024 (*n* = 20)	8	0	40%
2024–2025 (*n* = 18)	3	1	22%
Relative Risk			0.44

**Table 2:** Phases of the corrective exercise program and schedule of testing timepoints, as depicted by the respective team’s collegiate swimming schedule for the 2024–2025 season.

Season Timeline	Study Timeline	Week Number
First half of season Training block #1	Pre-season testing (PRE)	1
	Phase 1 instruction	2
	Phase 1	3
		4
	Phase 2 instruction	5
	Phase 2	6
		7
	Phase 3 instruction	8
	Phase 3	9
		10
Mid-season competition	Mid-season rest (repeat Phase 1)	11
	Mid-season competition (no exercises completed)	12
	Mid-season testing (MID)	13
Second half of season Training block #2	Phase 1.2 instruction	14
	Phase 1.2	15
		16
Holiday break	Optional at-home protocol	17
		18
Continuation of training block #2	Phase 2.2 instruction	19
	Phase 2.2	20
	Phase 3.2 instruction	21
	Phase 3.2	22
Conference championships (end of season competition)	End of season competition rest (repeat Phase 1.2)	23
		24
	End of season competition	25
	Post-season testing (POST)	26

**Table 3:** Corrective exercise program, with sets and repetitions, used for the duration of the study.

Exercise		Sets	Repetitions (Week 1, 2, 3)
Phase 1			
A1	4-point shoulder taps	2	5 PS, 6 PS, 8 PS
A2	Banded Cuban press	2	8, 10, 12
A3	Cross-body single-arm lat pulldown	2	5 PS, 6 PS, 8 PS
B1	Push-up to T-spine open	2	3 PS, 4 PS, 5 PS
B2	Prone V-raise isohold	2	30 s, 35 s, 40 s
B3	Prone W to T raise	2	5 PS, 6 PS, 8 PS
Phase 2			
A1	Banded single-arm overhead hold	2 PS	20 s, 25 s, 30 s
A2	Glute bridges with chin tuck	2	6, 8, 10
A3	Push-up plus	2	4, 5, 6
B1	Dumbbell shoulder external rotation	2	8, 10, 12
B2	Banded upright row	2	6, 8, 10
B3	Cross-body single-arm raises	2	5 PS, 6 PS, 8 PS
Phase 3			
A1	3-way band pullapart	2	3 PS, 4 PS, 5 PS
A2	Dumbbell side-lying external rotation	2	3 PS, 4 PS, 5 PS
A3	Face pulls	2	5, 6, 8
B1	4-way plank taps	2	3 PS, 4 PS, 5 PS
B2	Dumbbell pullover to serratus stretch	2	3 PS, 4 PS, 5 PS
B3	Forearm wall slides	2	5, 6, 8
Mid-Season Rest Week			
A1	Banded cross-body single-arm raise	2	6 PS, 4 PS
A2	Dumbbell Cuban press	2	8, 6
A3	Prone W to T raise	2	6, 4
B1	Glute bridges with chin tuck	2	8, 6
B2	Dumbbell side-lying external rotation	2	6 PS, 4 PS
B3	Push-up plus to plank toe taps	2	4 PS, 3 PS
Phase 1.2			
A1	Child’s pose	1	:45 s
A2	Partner lat stretch	1 PS	:30 s
A3	Thread-the-needle stretch	1 PS	:30 s
B1	4-way plank taps	1	3 PS, 4 PS, 5 PS
B2	Cross-body single-arm raises	1	5 PS, 6 PS, 8 PS
B3	Banded upright row	1	6, 8, 10
Phase 2.2			
A1	Standing pec stretch	1 PS	:30 s
A2	Supine upper-trap tennis ball rolling	1 PS	:60 s
A3	Dumbbell bent hanging-arm circles	1 PS	10 clockwise, 10 counter
B1	Dumbbell side-lying external rotation	1	6 PS, 8 PS
B2	Dumbbell Cuban press	1	8, 10
B3	Push-up to T-spine open	1	3 PS, 4 PS
Phase 3.2			
A1	Thread-the-needle stretch	1 PS	:30 s
A2	Supine upper-trap tennis ball roll	1 PS	:60 s
A3	Partner lat stretch	1 PS	:30 s
B1	Cross-body single-arm raises	1	6 PS, 8 PS
B2	4-way plank taps	1	4 PS, 5 PS
B3	Banded upright row	1	6, 8
Conference Championship Rest Week			
A1	Standing pec stretch	1 PS	:30 s
A2	Supine upper-trap tennis ball roll	1 PS	:60 s
A3	Child’s pose	1	:45 s
B1	Banded bicep curl	1	8, 6
B2	Plank to toe taps	1	4 PS, 3 PS
B3	Dumbbell Cuban press	1	6, 4

Note: PS = per side; s = seconds.

**Table 4:** Mean and standard deviations of all measurements related to the CKCUEST, as well as significance and effect size.

Measure	PRE Mean ± SD	MID Mean ± SD	POST Mean ± SD	Sig. (*p*)	Effect Size (*d*)
Tap Count	24.10 ± 3.66 *	27.65 ± 4.42 *	28.96 ± 4.14 *	<0.01	0.67
Normalized Performance	0.36 ± 0.05 *	0.41 ± 0.06 *	0.43 ± 0.06 *	<0.01	0.67
Power	76.00 ± 18.26 *	87.35 ± 21.93 *	91.14 ± 19.73 *	<0.01	0.68

Note: Sig. = significance, * = Bonferroni corrections revealed significant increases from PRE to MID, MID to POST, and PRE to POST. For more details, view Section 3.

**Table 5:** Mean and standard deviations of all goniometric measurements collected at each laboratory testing timepoint, as well as differences between the two shoulders’ range of motion and the frequency (*n*) of Glenohumeral Internal Rotation Deficit (GIRD).

Shoulder Location	PRE (°) Mean ± SD	MID (°) Mean ± SD	POST (°) Mean ± SD	Sig. (*p*)	Effect Size (*d*)
Internal Rotation					
Left	74.56 ± 7.73	71.11 ± 5.20 *	99.39 ± 12.80 **	<0.01	0.89
Right	67.33 ± 10.69	74.94 ± 8.28 *	98.06 ± 14.38 **	<0.01	0.81
Difference	10.00 ± 5.62	6.50 ± 7.21	12.56 ± 8.86	0.08	0.27
External Rotation					
Left	93.61 ± 6.90	94.67 ± 9.78	96.94 ± 12.09	0.55	0.07
Right	94.39 ± 9.42	93.44 ± 9.52	96.22 ± 9.78	0.47	0.09
Difference	7.33 ± 5.91	7.78 ± 8.23	7.06 ± 6.78	0.92	0.01
Total Range of Motion					
Left	168.17 ± 10.08	165.78 ± 10.87 *	196.33 ± 20.17 **	<0.01	0.74
Right	161.72 ± 10.06	168.39 ± 11.35 *	194.28 ± 17.86 **	<0.01	0.84
Difference	10.44 ± 8.00	10.83 ± 14.30	11.94 ± 9.45	0.89	0.01
GIRD					
	PRE (*n*)	MID (*n*)	POST (*n*)	Sig. (*p*)	-
	1	1	3	0.45	

Note: Sig. = significance; GIRD = Glenohumeral Internal Rotation Deficit; * = significant difference from MID to POST seen through Bonferroni correction (details in Section 3); ** = significant difference from PRE to POST seen through Bonferroni correction (details in Section 3).

**Table 6:** Frequency (*n*) of positive clinical test results across the three-laboratory testing timepoints.

Test	Shoulder Location	PRE	MID	POST	Sig. (*p*)	Effect Size (*d*)
**Clinical Testing (*n*)**						
Hawkins-Kennedy	Left	2	4	2	0.51	-
	Right	3	4	2	0.61	-
Neer’s Sign	Left	1	0	1	0.61	-
	Right	1	0	0	0.37	-
Sulcus Sign	Left	0	0	0	N/A	-
	Right	0	0	0	N/A	-

Note: Sig. = significance.

## Data Availability

The data presented in this study may be available upon request from the corresponding author due to privacy reasons.

## Abbreviations

The following abbreviations are used in this manuscript:

NCAA	National Collegiate Athletic Association
ER	External rotation
IR	Internal rotation
ROM	Range of motion
CKCUEST	Closed kinetic chain upper-extremity stability test
TRM	Total range of motion
GIRD	Glenohumeral internal rotation deficit
